# Feasibility of whole body vibration during intensive chemotherapy in patients with hematological malignancies – a randomized controlled pilot study

**DOI:** 10.1186/s12885-018-4813-8

**Published:** 2018-09-25

**Authors:** Antonia Pahl, Anja Wehrle, Sarah Kneis, Albert Gollhofer, Hartmut Bertz

**Affiliations:** 1 Department of Medicine I (Specialties: Hematology, Oncology, and Stem-Cell Transplantation), Faculty of Medicine, Medical Center – University of Freiburg, Hugstetterstr. 55, 79106 Freiburg, Germany; 2 Institute for Exercise- and Occupational Medicine, Faculty of Medicine, Medical Center – University of Freiburg, Freiburg, Germany; 3 Department of Sport and Sport Science, University of Freiburg, Freiburg, Germany

**Keywords:** Exercise therapy, Postural balance, Cancer therapy, Acute leukemia, Bone marrow neoplasm, Intervention study, Activities of daily living

## Abstract

**Background:**

Hospitalized cancer patients undergoing intensive or high-dose chemotherapy often experience a considerable decline in functional performance associated with the increased risk of adverse health events. Exercises, particularly resistance-based exercises that may counteract this decline are restricted by therapy-related side effects. Since whole body vibration (WBV) is known to efficiently stimulate the neuromuscular system without significantly raising blood pressure, we hypothesize that especially WBV is particularly feasible even during intensive or high-dose chemotherapy (primary endpoint) and thus induces beneficial functional adaptations.

**Methods:**

Twenty hospitalized patients with hematological malignancies scheduled for intensive or high-dose chemotherapy were randomly allocated to an intervention group (IG) undergoing WBV, or an active control group (CG) cycling. Feasibility was determined by comparing the IG’s and CG’s training compliance. Furthermore, to assess feasibility, WBV-induced changes in chemotherapy-related side effects, blood pressure, and heart rate immediately after exercising were documented. To assess patients’ functional performance, we measured jump height (cm), the duration (sec) of performing the chair rising- (CRT) and timed-up-and-go test (TUG), maximum power output during jumping and CRT (watt/kg) as well as sway path (mm) during balance tasks.

**Results:**

Training compliance was similar between groups (IG: median 62%, range 39–77; CG: 67%, 58–100; *p* = 0.315). Moreover, we observed neither the IG’s reported side effects worsening, nor any increase in blood pressure after WBV. IG’s jump height (+ 2.3 cm, 95%CI 0.1–4.4, *p* = 0.028) and TUG performance (− 1.3 s, 95%CI -2.53 – -0.65, *p* = 0.027) improved significantly, while sway paths in semi-tandem stance were augmented after the intervention (eyes open: + 60 mm, 95%CI 2–236, *p* = 0.046; eyes closed: + 88 mm, 95%CI 49–214, *p* = 0.028). The CG’s performances did not change over time. Maximum power output during CMJ and CRT and time during CRT did not change.

**Conclusion:**

Our study is the first proving the feasibility of WBV during intensive/high-dose chemotherapy of hospitalized cancer patients. Additionally, WBV-induced neuromuscular adaptations resulted in functional benefits relevant to patients’ autonomy. We believe that WBV can be implemented as an alternative training method during intensive chemotherapy, although the relative benefit compared to conventional resistance training requires more evaluation in future studies.

**Trial registration:**

German Register of Clinical Trials No.: DRKS00004338, prospectively registered on 11/30/2012.

## Background

Many patients diagnosed with hematologic malignancies (e.g. leukemia) undergo intensive or high-dose chemotherapy [1, 2] leading to cytopenia and other side effects [3, 4]. These are manifold and can impair physical and psychological functioning considerably. Because of the growing risk of complications due to aplasia (e.g. infections, bleeding), patients are usually hospitalized for two to 4 weeks, meaning a period of restricted mobility associated inter alia with muscle-mass loss, and reduced strength capacity and functional performance [5, 6]. It is common knowledge that impaired functional performance and poor body composition, i.e. disproportionally less muscle mass, limit patients’ autonomy and quality of life [7, 8] and may raise the mortality risk [9–11]. All cancer patients are therefore recommended to be physically active during treatment while considering specific limitations to counteract physical deconditioning and preserve mobility [12–14]. There is evidence that exercising (e.g. aerobic exercises, especially cycling) even during high-dose chemotherapy (as conditioning therapy prior to allogeneic hematopoietic cell transplantation) is safe and effective, and has the potential to reduce physical and psychosocial side effects [13, 15–19]. However, aerobic exercises alone appear insufficient to affect functional impairments especially, whereas some studies added resistance training to their intervention program [19–21]. They confirmed the feasibility of resistance training during chemotherapy and demonstrated positive effects on patients’ physical functioning, but could not entirely maintain muscle strength during hospitalization. Taking into account catabolic medication [22, 23] and the amount of time patients are exposed to bed rest, high intensities and volumes of resistance training were needed to significantly avoid losing strength [24] and its functional consequences, factors that are relevant prognostic factors for survival [11]. However, according to current recommendations [14, 25] patients’ blood values, i.e. platelets counts, and well-being may limit the intensity and volume of resistance training patients are able to perform during intensive chemotherapy.

We therefore planned to introduce whole body vibration (WBV) as a resistance training method for patients undergoing intensive chemotherapy during hospital stay. WBV induces frequency-dependent repeating contractions of the muscles culminating in a tonic vibration reflex that enhances the recruitment of motor units [26, 27]. WBV thus reveals EMG activity similar to that during conventional squat exercises with external loads [28]. It thus stimulates the musculoskeletal system very time-efficiently and can improve muscle strength [29] and functional performance [30]. Furthermore, it evidently prevents muscle-mass loss during bed rest [6, 31]. With all these effects, WBV has little impact on vital parameters, e. g. blood pressure [32, 33]. We therefore believe that WBV is feasible even for patients during thrombocytopenia. Additionally, it might be less tiring and easier to carry out, but similarly effective to resistance training because of the high neuromuscular activity even under passive conditions when following current recommendations [14]. Up to now, results of WBV’s feasibility or effectiveness are only available to cancer patients after active anti-cancer treatment [34–36]. There is only one ongoing study [37] investigating WBV during chemotherapy, but no results have yet been published. We thus implemented a randomized controlled pilot study to assess primarily the feasibility of WBV during intensive and high-dose chemotherapy during hospital stay. Since aerobic exercise has proven to be safe and feasible for hospitalized patients during high-dose chemotherapy [16, 38] we aimed to compare exercise’s feasibility of WBV versus an active control group performing aerobic cycling exercises. Secondarily, we attempted to evaluate WBV-induced effects on patient’s functional performance. We hypothesized that patients would adhere to the WBV exercise sessions as prescribed despite therapy-related side effects without exhibiting differences from the control group. Furthermore, WBV might induce neuromuscular adaptations leading to functional benefits.

## Methods

### Study design and patients

The main objective of this randomized controlled pilot study was to confirm WBV’s feasibility in hospitalized patients with hematological malignancies undergoing intensive or high dose chemotherapy. Patients were recruited at the Department of Medicine I, University Medical Center Freiburg, Germany, immediately after hospital admission. Within a 7-month time frame, eligible patients were randomly allocated to either an intervention group (IG) or active control group (CG). Block randomization was based on a computer-assisted pseudo-random number generator (Research Randomizer, Version 4.0). Inclusion criteria were diagnosis of a hematological malignancy, scheduled for intensive/high-dose chemotherapy with a two- to four-week period of hospitalization, and written informed consent. Absolute exclusion criteria were any unstable bone metastasis, a knee or hip endoprosthesis, epilepsy, pacemaker, severe cardiovascular diseases [36, 39] and threshold blood-count values below safety criteria (platelets count ≤10.000/μl and hemoglobin ≤8 g/dl) [40, 41]. Relative exclusion criteria included stents or former joint injuries. Baseline parameters were assessed before the first administration of chemotherapy (T0) and post-evaluated immediately before discharge (T1), respectively. Table 1 summarizes patients’ clinical information. The study was approved by the Ethics Committee of the University of Freiburg and conducted according to the Declaration of Helsinki (German Register of Clinical Trials No.: DRKS00004338).

**Table 1:** Clinical information of completer (*n* = 11) and all randomized patients (*n* = 20, completer and drop-outs)

	Completer		All
	IG *n* = 6	CG *n* = 5	n = 20
Age [years]^a^	47 (19–62)	56 (32–63)	55 (47–63)
Sex [n] male:female	5:1	3:2	14:6
BMI [kg/m^2^]^a^	26 (20–28)	26 (22–28)	26 (25–27)
Diagnosis [n]			
AML	1	4	8
ALL	1	0	1
APL	1	0	1
NHL	2	0	5
HL	0	0	1
t-cell lymphoma	1	0	1
MW	0	0	1
MM	0	1	1
PMF	0	0	1
Remission [n]			
ED	1	3	7
SD	0	1	2
PD	1	0	1
CR	0	1	2
PR	0	0	1
recurrence	2	0	5
N/A	2	0	2
Time since initial diagnosis [weeks]^a^	46 (2–371)	6 (2–215)	32 (7–128)
Cycles of chemotherapy before admission [n]^a^	9 (0–16)	1 (0–9)	4 (1–9)
Type of chemotherapy during intervention [n]			
High-dose prior to autologous HSCT	1	1	5
Induction therapy for AML	4	3	8
Intensive chemotherapy for ALL, HL, NHL	1	1	7
Duration of hospitalization [days]^a^	27 (21–56)	27 (16–69)	26 (22–34)
Karnofsky performance index [%]^a^	95 (80–100)	90 (80–100)	90 (85–90)

^a^Median (range), *BMI* body mass index, *AML* acute myeloid leukemia, *ALL* acute lymphocytic leukemia, *APL* acute promyelocytic leukemia, *NHL* non-Hodgkin lymphoma, *HL* Hodgkin lymphoma, *MW* morbus Waldenström, *MM* multiple myeloma, *PMF* primary myelofibrosis, *ED* initial diagnosis, *SD* stable disease, *PD* progress disease, *CR* complete remission, *PR* partial remission, *HSCT* hematopoietic cell transplantation

### Intervention

Both groups’ one-on-one training sessions took place in a separate room on the patients ward during three sessions per week, each lasting 20 min.

The IG’s exercise protocol included three sets of two to four different exercises (static and dynamic squats, heel raise and combination of both) on the Galileo® Sport vibration platform (Novotec Medical GmbH, Pforzheim, Germany) which is adjustable in steps of 0.5 Hz within a range of 5–30 Hz. Each exercise lasted 30 to 60s depending on patients’ condition with a 30 to 60s rest between exercises and 60 to 120 s between sets. Patients should reach intensity prescription of 14 to 16 on the perceived exertion rating scale (Borg 6–20) [40, 42]. To generate the best neuromuscular response, exercises were performed within a frequency range of 18–25 Hz and at 3.5-4 mm amplitude [43, 44]. During static exercises, patients were asked to shift their body weight on their forefeet and to maintain a knee angle of approx. Sixty degrees flexion in static position [43]. If patients couldn’t maintain the forefoot position throughout the exercise period, we supported their stance position via a heel foam wedge.

The CG performed aerobic exercises on a bicycle ergometer. Exercise sessions also lasted up to 20 min with the same intensity prescription as the IG. For patients unable to endure 20 min continuous cycling, an individual interval program with periods of rest was used to achieve 20 min cycling in total for each exercise session.

We consulted patients’ files for blood values before each exercise session to ensure their values met our safety criteria: platelets count ≥10.000/μl and hemoglobin ≥8 g/dl. Additionally, we controlled patients’ blood pressure and heart rate before and after each exercise session to avoid overload. Blood values, blood pressure, training progress and reasons for missed sessions were documented.

### Outcome measures

#### Primary endpoint feasibility

We determined the feasibility of WBV by comparing the IG’s and CG’s training compliance. Training compliance in percent was calculated as completed exercise sessions divided by planned exercise sessions.

Furthermore, exercise-related adverse events were documented and reasons for missed exercise sessions were compared between groups. The tolerability of vibration training was also assessed via a self-designed questionnaire that asked (a) for the extent of chemotherapy-induced side effects (concerning pain, illness, dizziness, etc.) immediately before and after each vibration training session to examine whether WBV might enhance or weaken these side effects and (b) to determine their WBV-induced well-being. This questionnaire included 18 items scored from 0 to 3 (not at all, marginally, quite a lot, very much). Additionally, blood pressure and heart rate were measured and documented immediately before and after exercising as well as at the two measurement sessions (T0 and T1).

#### Secondary endpoint functional performance

All functional performance measurements were taken on a force plate (Leonardo Mechanograph® GRFP, Novotec Medical GmbH, Pforzheim, Germany) that determined dynamic ground reaction forces in its local and temporal progress. Data were recorded with a sample rate of 800 Hz and analyzed using Leonardo Mechanograph® Research-Software (Novotec Medical GmbH, Pforzheim, Germany).

To test functional performance under dynamic conditions, two common functional tasks were performed: a maximum counter-movement jump (CMJ) and a chair-rising test (CRT). Both tests evaluate leg muscle power output which in turn reflects the efficiency of lower limb neuromuscular interaction [45]. Patients executed CMJ with freely-moving arms and were instructed to jump as high as possible. Outcomes were defined as maximum power output during take-off per kilogram body weight (P_max_CMJ_; W/kg) and jumping height (cm). For CRT, patients started in a sitting position with their arms crossed over their chest. Patients had to stand up from a bench (45 cm, Novotec Medical GmbH) and sit down again five times in a row. They were asked to rise up until full extension of the knee and hip and to sit down using their full bodyweight. Durations (sec) of the second, third and fourth repetition were recorded and the average duration was calculated of one repetition [46]. Additionally, power output during stand up (P_max_CRT;_ W/kg) was measured for the CRT. Best values out of two trials per test condition were used to analyze CMJ and CRT.

Functional performance under static conditions was evaluated by changes in balance performance, known to indicate neuromuscular and functional impairments [47, 48]. We determined the center of force (COF) displacement in anterio-posterior and medio-lateral direction during three different stance conditions: semi-tandem stance with eyes open (EO) and eyes closed (EC) and one-leg stance in EO condition. Assessments took place without shoes. Patients were asked to stand upright and comfortably and direct their gaze onto a marked spot located at eye level on the wall. Sway path (mm) and standard ellipse (cm^2^) of COF was recorded over a period of 30s. The mean value out of three trials was used for analysis.

#### Secondary endpoint mobility

To assess basic functional mobility in a daily situation, we used the timed-up-and-go test (TUG) commonly used to evaluate patients’ autonomy in daily life [49]. Duration (sec) was recorded from the moment patients got up from an armed chair, walked three meters, turned around, walked back and sat down on the chair again [49, 50].

### Statistics

All variables were included in non-parametric analysis as the assumption of normal distribution (Shapiro-Wilk test) was not satisfied and the sample size is small. Differences in training compliance between both groups and differences of groups’ delta (T1-T0) were assessed by Mann-Whitney-U-test. Intragroup differences over time were computed by Wilcoxon signed-rank test. The level of significance was set to *p* < 0.05. Group data are presented as median and 95% confidence interval (95% CI). To estimate the treatment effect the point estimate and 95% confidence interval of the Hodges-Lehmann’s median differences for paired groups were used. All statistical analyses were conducted using IBM SPSS Version 22 software (SPSS Inc., Chicago, Illinois, USA).

## Results

**Fig. 1:**
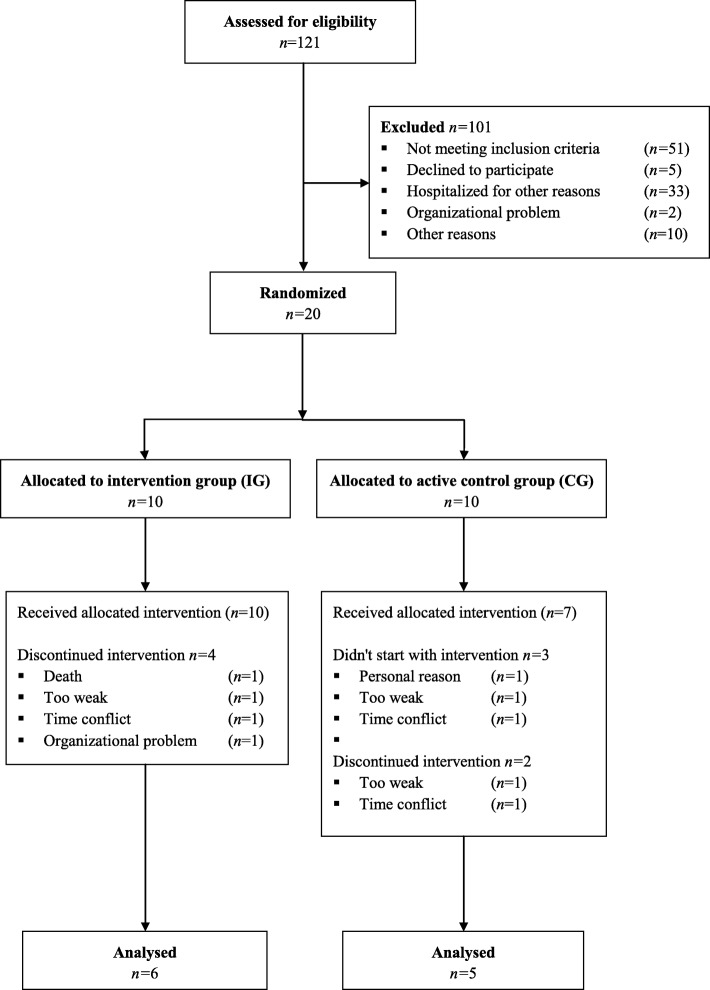
Flow diagram of study recruitment

No adverse events were observed during the study period. No baseline assessment was possible in three CG patients. Thus 17 patients participated in our intervention program and were included in the feasibility analysis. However, no post-intervention data on two CG and four IG patients were available (Fig. 1). Eleven patients were thus ultimately included in our functional performance and mobility analyses.

### Feasibility

**Fig. 2:**
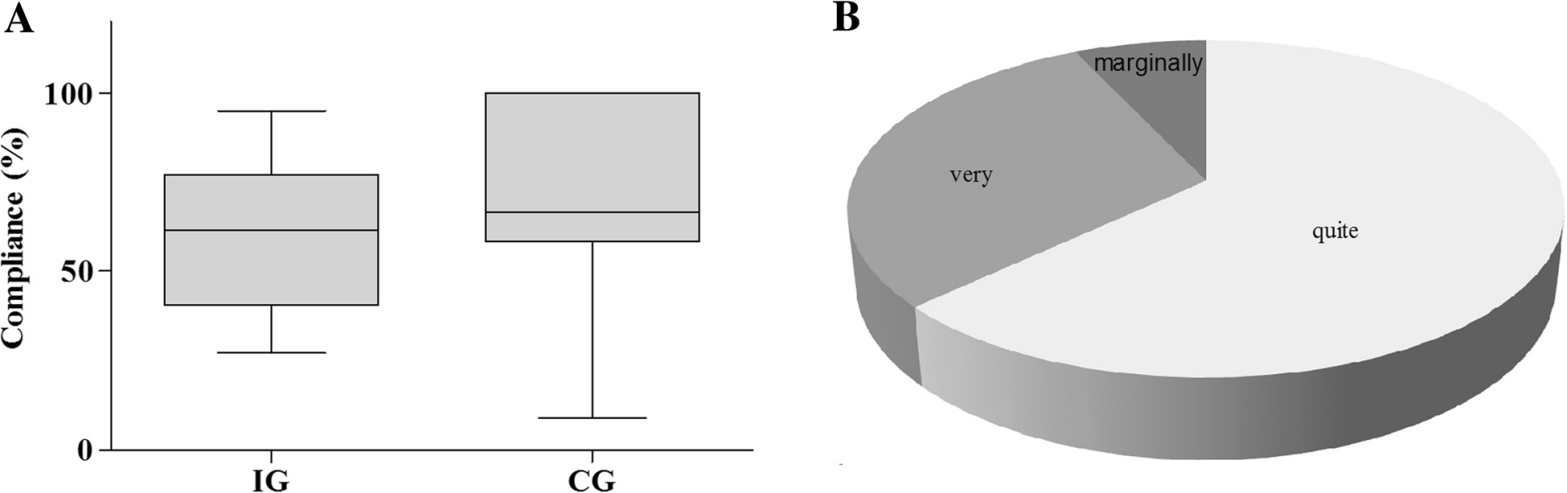
Feasibility of WBV - Rate of training compliance and individual training perception. **a** Distribution of training compliance for IG and CG. Box-and-whisker plots showing the lower quartile (25th percentile), median (50th percentile), upper quartile (75th percentile) and degree of dispersion as 95% confidence interval (95% CI). **b** Percentage distribution of individual training’s perception after WBV following the question “Did the exercise session do you any good?/Did the workout make you feel good?”. Quite good 63.27%, very good: 29.97%, marginally good 6.66%, not good at all 0%

No adverse events or differences in groups’ training compliance were observed during any of the WBV training sessions (IG: 62%, CG: 67%, *p* = 0.315) (Table 2 and Fig. 2a). Reasons for missed exercise sessions were similar between groups (Table 2) and no training session had to be cancelled due the type of exercise. Furthermore, WBV training did not worsen subjective chemotherapy-induced side effects: − 0.02 (a difference of 0 would represent no change in chemotherapy-induced side effects after training compared to pre-training, while 3 would represent a great increase). Additionally, 63% of IG patients experienced WBV as “quite good”, 30% as “very good”, 7% as “marginally good” and 0% feedback “not good at all” (Fig. 2b). We observed no changes in either group in their blood pressure or heart rate values post-exercising compared to pre-exercising as well as post-intervention compared to pre-intervention (Table 3).

**Table 2:** Comparison of groups’ training compliance and reasons for missed exercise session for all patients participating in the training program (*n* = 17)

	IG *n* = 10	CG *n* = 7	P
Compliance [%]^a^	62 (39 77)	67 (58–100)	0.315
Completed exercise sessions [n]^a^	5 (2–6.5)	6 (2–8)	0.417
Reason for missed exercise sessions			
Blood values [n]	9	2	0.109
Well-being [n]	17	9	0.475
Infection [n]	1	4	0.536
Reduced vigilance^b^ [n]	13	7	1.000
Afraid of worsened side effects [n]	1	3	0.887
Another appointment [n]	0	1	0.669
Separate room for training was not available [n]	1	0	0.740

^a^Median (range), *n* number of missed exercise sessions; ^b^patients were unresponsive

**Table 3:** Changes in vital parameters and weight

		*n*	T0^a^ median (range)	*n*	T1^a^ median (range)	median difference (95% CI)	*P*
Blood pressure							
Systolic (mmHg)	IG	6	128 (110–145)	6	110 (100–123)	−20 (− 43–0)	0.068
	CG	5	120 (115–130)	5	110 (105–125)	− 8 (− 20–5)	0.141
	P		0.662		0.537	0.662	
Diastolic (mmHg)	IG	6	80 (70–90)	6	70 (70–80)	−8 (− 15–3)	0.102
	CG	5	80 (60–85)	5	75 (70–90)	0 (− 5–15)	0.705
	P		0.662		0.247	0.177	
Heart rate (bpm)	IG	6	81 (64–97)	6	94 (71–100)	11 (− 11–22)	0.345
	CG	5	92 (76–100)	5	80 (76–90)	−5 (− 22–13)	0.225
	P		0.329		0.537	0.177	
Weight (kg)	IG	6	76 (67–80.3)	6	74.4 (66–83.5)	0.1 (−2.5–3.2)	0.917
	CG	5	80 (71.8–91)	5	77 (73–80)	−2.2 (− 12–1.2)	0.144
	P		0.247		0.662	0.247	
		*n*	Before exercise session^a^ median (range)	*n*	After exercise session median (range)	median difference (95% CI)	*P*
Blood pressure							
Systolic (mmHg)	IG	6	118 (112–137)	6	118 (113–135)	0.5 (−4–3)	0.686
	CG	5	114 (112–127)	5	119 (111–136)	4 (− 1–9)	0.078
	P		0.792		1.000	0.082	
Diastolic (mmHg)	IG	6	79 (68–86)	6	78 (73–83)	0 (− 3–5)	0.893
	CG	5	76 (71–82)	5	77 (71–84)	1 (0–3)	0.068
	P		0.792		0.931	0.429	

^a^Median (range); *bpm* beats per minute

### Functional performance

IG patients improved their jump height significantly (2.3 cm, 95% CI 0.1–4.4, *p* = 0.028), while the CG’s jump height was unchanged (− 3.3 cm, 95% CI -13.0–3.6, *p* = 0.345). Power output (P_max___CMJ_) of both groups remained stable over time. No changes after intervention were observed either in any group (IG or CG) for the duration of CRT or in their power output during CRT (P_max_CRT_).

During balance tasks, only the IG’s sway path in the semi-tandem stance conditions was augmented after intervention (EO: 60 mm, 95% CI 2–236, *p* = 0.046; EC: 88 mm, 95% CI 49–214, *p* = 0.028). The monopedal stance revealed no changes. All the CG’s balance performances were unchanged. Furthermore, our analysis of standard ellipse revealed no inter- or intra-group changes. All functional-performance results are illustrated in Table 4.

**Table 4:** Results of functional performance and mobility

		*n*	T0 median (range)	*n*	T1 median (range)	median difference^a^ (95% CI)	*P*
Functional performance							
Jumping height (cm)	IG	6	24.9 (24.5–45.0)	6	28.0 (26.1–45.2)	2.3 (0.1–4.4)	0.028*
	CG	5	25.6 (20.6–36.5)	5	24.4 (18.3–33.1)	− 3.3 (−13.0–3.6)	0.345
	P		0.792		0.329	0.126	
P_max___CMJ_ (W/kg)	IG	6	26.3 (23.5–44)	6	25.3 (25–45.3)	− 0.9 (−3.9–1.4)	0.345
	CG	5	28.1 (24.8–28.1)	5	28.6 (20.9–30.8)	− 0.6 (− 14.5–2.7)	0.500
	P		0.662		0.792	1.00	
Duration_CRT (sec)	IG	6	1.9 (1.2–2.8)	6	1.8 (1.1–2.9)	−0.0 (− 0.2–0.2)	0.686
	CG	5	2.5 (1.9–3.4)	5	2.5 (1.5–3.7)	− 0.0 (− 0.6–0.6)	0.686
	P		0.247		0.429	1.00	
P_max___CRT_ (W/kg)	IG	6	10.9 (8.9–17.7)	6	11.4 (9.6–18.6)	−0.1 (−1.1–1.3)	0.600
	CG	5	12.4 (8.1–14.2)	5	10.9 (6.8–16.4)	0.1 (−3.3–3.1)	0.893
	P		0.792		0.792	1.00	
Balance performance							
ST_EO_ sway path (mm)	IG	6	549 (447–591)	6	579 (457–713)	60 (2–236)	0.046*
	CG	5	498 (333–794)	5	471 (390–585)	−47 (− 220–128)	0.225
	P		0.249		0.247	0.082	
ST_EO_ standard ellipse (cm^2^)	IG	6	3.6 (1.6–8.0)	6	4.4 (2.8–6.8)	0.9 (−1.1–2.3)	0.345
	CG	5	2.3 (0.8–7.7)	5	3.6 (0.8–4.4)	0.3 (−1.5–2.9)	0.893
	P		0.249		0.247	0.622	
ST_EC_ sway path (mm)	IG	6	1117 (656–1386)	6	1203 (668–1503)	88 (49–214)	0.028*
	CG	5	888 (13–2171)	5	935 (655–1672)	−142 (−500–122)	0.345
	P		0.792		0.429	0.126	
ST_EC_ standard ellipse (cm^2^)	IG	6	9.9 (3.2–12.2)	6	9.4 (2.5–11.3)	0.5 (−3.2–3.2)	0.753
	CG	5	5.1 (2.7–11.5)	5	5.2 (1.7–9.1)	−0.9 (−2.4–0.6)	0.225
	P		0.177		0.126	0.931	
MS_EO_ sway path (mm)	IG	6	1621 (1295–2350)	4	1360 (926–2462)	−125 (− 482–176)	0.465
	CG	5	1394 (1264–2111)	5	1546 (1254–1731)	−53 (− 379–273)	0.500
	P		0.429		0.556	1.00	
MS_EO_ standard ellipse (cm^2^)	IG	6	8.0 (6.6–16.9)	4	5.3 (3.6–8.1)	−3.2 (−11.6 – −0.6)	0.068
	CG	5	6.1 (3.9–8.3)	5	5.5 (3.8–8.8)	−0.1 (−1.1–1.5)	0.500
	P		0.177		0.730	0.063	
Mobility (TUG)							
Duration (sec)	IG	6	6.2 (3.7–10)	6	5 (2.9–7.7)	−1.3 (−2.5 – −0.7)	0.027*
	CG	5	5.9 (4.5–17)	5	5.5 (4.8–10.6)	−1.1 (−6.4–1)	0.138
	P		0.662		0.329	1.00	

*indicates a significant difference (*p* < 0.05); ^a^prescribes the treatment effect by point estimation and 95% confidence interval of the Hodges-Lehmann’s median differences for paired groups; *ST*_*EO*_ semi-tandem stance with eyes open, *ST*_*EC*_ semi-tandem stance with eyes closed, *MS*_*EO*_ monopedal stance with eyes open, *MS*_*EC*_ monopedal stance with eyes closed, *TUG* timed-up-and-go test

### Mobility

IG patients reduced the time they needed to do the TUG test (− 1.3 s, 95% CI -2.53 – -0.7, *p* = 0.027), while the CG revealed no change (− 1.1 s, 95% CI -6.4 – 1, *p* = 0.138) (see Table 4).

## Discussion

Aim of our prospective, randomized controlled pilot study was to prove for the first time the feasibility of WBV training for hospitalized cancer patients during intensive or high-dose chemotherapy. We did not observe any WBV-related adverse events, the groups’ training compliance and reasons for missed exercise sessions were similar, and patients did not report any worsening of chemotherapy-related side effects after WBV sessions. Furthermore, as previously assumed, blood pressure was nearly unaffected by WBV. We thus succeeded in proving the feasibility of WBV in this setting and have justified its application in subsequent investigations evaluating its relative benefits. Additionally, we detected functional benefits due to WBV, indicated by their improved jumping- and TUG performance. Although our intervention did not lead to significant differences between groups over the intervention period, our results indicate that WBV may counteract some functional worsening during chemotherapy.

Due to the loss of physical capacity during treatment [51, 52] cancer patients are encouraged to be physically active to preserve their functional status [12, 14] and to reduce the risk of adverse health outcomes [11]. Aerobic exercise is well-researched, and it is safe and effective for cancer patients even during most intensive treatments [16, 38]. However, in light of muscle-mass loss and a considerable decline in functional performance [18], intensive and functional-oriented resistance exercises might counteract those impairments more effectively than aerobic exercises. In general, a patient’s condition and potential therapy-related side effects like cytopenia i.e. thrombocytopenia will undoubtedly limit the intensity and volume of resistance training, especially in those undergoing intensive chemotherapy [14]. Thus, resistance training during chemotherapy usually consists of exercises with therabands and body weight in combination with aerobic exercises [19, 53, 54]. To date, there is only one randomized controlled study [21] investigating the effect of exclusively resistance training on cancer patients during chemotherapy. However, that study focused solely on the effects on protein metabolism and did not analyze functional performance [21]. Resistance training with high intensity and great neuromuscular impact would be most effective to counteract muscle mass loss during long periods of inactivity [55]. Indeed, this is associated with a rise in blood pressure [56, 57] that increases the risk of bleeding when patient’s platelets are low. Furthermore, it usually requires that participants be willing to actively exhaust themselves in a state of physical discomfort. Thus, considering a patient’s physical and mental condition in line with current guidelines [14, 40], high intensity resistance training during intensive chemotherapy is not feasible. On the other hand, WBV is a strengthening exercise method known to increase muscle power output even under passive training conditions by inducing a tonic-vibration reflex addressing many muscle fibers simultaneously [26, 29]. Additional exercises like the static and dynamic squats our patients also performed can further intensify muscular activity [58]. Furthermore, WBV-induced periodic dynamic movements cause increased blood flow, potentially inhibiting a rise in blood pressure [33]. Thus, WBV has less impact on vital signs [59, 60]. Since we documented blood pressure immediately before and after each WBV-exercise session, our results are in line with research findings indicating that WBV does not lead to a considerable increase in blood pressure. We therefore assume that WBV is a convenient and low-threshold exercise method for patients undergoing intensive chemotherapy and unable to perform exercises of high intensity and long duration. Accordingly, we are the first to have confirmed its feasibility despite chemotherapy-related side effects in this study. Patients were capable of performing the prescribed exercises during the 20-min WBV sessions, and none reported a worsening of physical well-being after their WBV session or complained of being overloaded by our program. Consequently, we observed good training compliance.

What is clinically relevant: WBV training positively influenced functional performance. In line with other working groups who observed improved strength capacity [61, 62] or improved jump height [44, 63] after WBV especially in weak or untrained persons, we detected enhanced jumping and TUG performance in our IG after the intervention. Jumping performance is influenced by various factors: the rate of force development, which in turn is affected by the stretch-shortening cycle’s efficacy [64, 65] and the level of reactive strength so essential for common daily situations such as adequate reactions to unexpected perturbations [66, 67]. WBV induces frequency-dependent, repeated muscle contractions culminating in a tonic vibration reflex that enhances the recruitment of motor units [26, 27] and that may improve the stretch-shortening cycle and inter- and intramuscular coordination. Reactive strength is more likely to depend on neuromuscular processes than on muscle size [68] and thus represents a clear functional gain. For example, reactive strength is an influencing variable of walking speed, one acknowledged predictor of mortality [9, 69, 70]. Consequently, enhanced jumping performance means improved interaction of muscles, tendons, and the neuromuscular system and therefore better functioning of the locomotoric system. The functional gains due to WBV are also visible in the improved performance on the TUG test. TUG is a common clinical test to evaluate patients’ mobility [71]. The time needed to complete this task strongly correlates with the independence during daily activities [46]. Thus, enhancing this ability is a crucial factor for a patient’s autonomy. CRT performance, also a common clinical test for daily activity function and lower body strength [71], remained stable on a level comparable to age-adjusted reference values [40, 46]. Taking into account that the IG and CG carried out five respectively six exercise sessions during 27 days of hospitalization, we assume that the amount and intensity of WBV exercise sessions were insufficient to significantly affect lower-body strength. But we believe that our WBV intervention could enhance neuromuscular interaction and inter- and intramuscular coordination, in line with findings of Perchthaler et al. (2015) [72]. In general, inter- and intramuscular coordination is enhanced prior to considerable increases in strength [73, 74]. Accordingly, we presume that a longer intervention period with consequently more exercise sessions would also reveal group effects over time. Moreover, we propose evaluating muscle strength and function in greater detail to confirm our preliminary findings. Overall, preserving or even improving functional performance should be of particular interest when exercising with cancer patients receiving medical treatment, because there is ample evidence that functional impairments are highly relevant prognostic factors for survival [11]. Factors such as long periods of inactivity [6, 31, 75], neurotoxic agents among chemotherapy [76] or aging itself [45] lead to difficulty connecting sensory information and motor action [77]. This often results in impaired postural control and balance performance - determinants of functional status and significant risk factors for falls [78, 79]. We speculated that the repetitive stimuli of external perturbation induced by WBV would positively affect posture stability [62, 80]. In this context, Streckmann et al. (2018) [37] are even investigating WBV’s preventive effect on neuropathic symptoms, including postural stability, in patients undergoing neurotoxic chemotherapy. However, against our expectations, our balance-control analysis revealed that WBV was not superior to aerobic exercise. During the semi-tandem stance after intervention, IG’s sway path was even augmented. According to two reviews, WBV’s effects on balance performance remain inconsistent [81, 82]. Muehlbauer et al. [83] defined balance control and lower-extremity strength as independent neuromuscular systems that should be exercised separately. In conclusion, we propose that specific balance exercises be included that may prevent or even reduce balance impairments during hospitalization and thus further lower the risk of adverse health events [84]. Considering the non-significant post-intervention group effects, we cannot explicitly assume WBV’s superiority to aerobic exercises. However, the functional improvements we observed exclusively in our IG support the hypothesis that WBV has greater influence on neuromuscular adaptions than aerobic cycling.

## Conclusion

To best of our knowledge, our prospective, randomized controlled pilot study is the first study proving the feasibility of WBV immediate during intensive or high-dose chemotherapy in hospitalized cancer patients. Our results also suggest that WBV can improve mobility and jumping height in these patients. Such factors might be associated with greater autonomy and even better survival prognosis. We thus recommend implementing WBV as an alternative training method to aerobic exercise training during intensive chemotherapy to maintain patients’ functional status. However, the specific advantages and relative benefits compared to other forms of resistance training require further investigation in greater detail and with larger sample sizes. The success we have had in our pilot study paves the way for further WBV-based investigations during chemotherapy.

## Abbreviations

CG: Control group
CI: Confidence interval
CMJ: Counter movement jump
COF: Center of force
CRT: Chair rising test
EC: Eyes closed
EO: Eyes open
IG: Intervention group
TUG: Timed-up-and-go
WBV: Whole body vibration
